# A Low Collision and High Throughput Data Collection Mechanism for Large-Scale Super Dense Wireless Sensor Networks

**DOI:** 10.3390/s16071108

**Published:** 2016-07-18

**Authors:** Chunyang Lei, Hongxia Bie, Gengfa Fang, Elena Gaura, James Brusey, Xuekun Zhang, Eryk Dutkiewicz

**Affiliations:** 1 School of Information and Communication Engineering, Beijing University of Posts and Telecommunications, Beijing 100876, China; leichunyang.2014@gmail.com (C.L.); zhangxuekun1990@gmail.com (X.Z.); 2 School of Computing and Communications, University of Technology Sydney, Sydney 2109, Australia; gengfa.fang@uts.edu.au (G.F.); Eryk.Dutkiewicz@uts.edu.au (E.D.); 3 Faculty of Engineering and Computing, Coventry University, Coventry CV1 5FB, UK; e.gaura@coventry.ac.uk (E.G.); james.brusey@gmail.com (J.B.)

**Keywords:** medium access control, carrier sensing range, data collection, wireless sensor network

## Abstract

Super dense wireless sensor networks (WSNs) have become popular with the development of Internet of Things (IoT), Machine-to-Machine (M2M) communications and Vehicular-to-Vehicular (V2V) networks. While highly-dense wireless networks provide efficient and sustainable solutions to collect precise environmental information, a new channel access scheme is needed to solve the channel collision problem caused by the large number of competing nodes accessing the channel simultaneously. In this paper, we propose a space-time random access method based on a directional data transmission strategy, by which collisions in the wireless channel are significantly decreased and channel utility efficiency is greatly enhanced. Simulation results show that our proposed method can decrease the packet loss rate to less than 2% in large scale WSNs and in comparison with other channel access schemes for WSNs, the average network throughput can be doubled.

## 1. Introduction

Wireless sensor networks (WSNs) play an important role in sensing and collecting a wide range of environmental and geological parameters in the current and future surveillance systems. In such networks, widely covered and densely deployed sensors may simultaneously detect the sensing data and they need to send the data to a sink node. As a result, the effective network throughput will be severely decreased because of the collisions among the contending sensors accessing the wireless channel simultaneously. With the increasing demand for high network converge and sensor density with new applications such as Internet of Things (IoT) and Vehicular-to-Vehicular (V2V) networks, the possibility of collisions among WSN nodes has become too high for WSNs to work well. Therefore, how to achieve effective data collection through an efficient channel access and scheduling mechanism so as to decrease the collisions becomes a new challenge in the new IoT and V2V types of super dense WSNs.

In Carrier Sense Multiple Access with Collision Avoidance (CSMA/CA) based networks, the transmission by hidden nodes causes severe interference, i.e., collision, to an on-going transmission in WSNs. In this paper, we focus on seeking a new method to avoid such interference.

So far, channel estimation based backoff algorithms [1,2] have been proposed to avoid collisions among the neighbor nodes. In such algorithms, nodes adaptively schedule their time to access the channel by continuously estimating the contention levels among their neighbor nodes. It has been shown in [3] that channel estimation based backoff algorithms can efficiently decrease collisions among neighbor nodes to the theoretical low bound in cases with a wide range of contention levels. However, such algorithms are invalid for the case of collisions from hidden nodes because collisions from hidden nodes are too complex and hard to monitor on-the-fly.

Decreasing the possibility of collisions from hidden nodes with as small throughput loss as possible is challenging. In recent years, the studies in [4,5,6] re-evaluated the Channel Clear Assessment (CCA) function and tried to solve the hidden terminal problem by proposing new solutions based on the new concept of efficient Carrier Sense Ranges (CSR). By enabling the non-hidden nodes to detect and hear the hidden nodes’ activities, the hidden nodes will no longer be hidden for others. Thus, a lot of interference analysis models have been proposed since then for better CSRs for different applications. The authors in [7,8,9,10] proposed Protocol Interference Models (PrIMs) to improve performance of CSRs in networks with diverse topologies. In their models, the interference introduced by a hidden node to an on-going communication is comprehensively analyzed, and a “safe” CSR is calculated to prevent the on-going communication from being corrupted by this hidden node. However, the work in [4] indicated that PrIMs is idealistic because the interference in reality is not from a single hidden node but from multiple hidden nodes. As a consequence, the CSRs calculated from PrIMs are not large enough to effectively avoid collisions caused by hidden nodes. A new interference analysis model, Physical Interference Model (PhIM), was proposed in [11,12]. In these papers, interferences from all possible hidden terminals are included so that the CSRs calculated from PhIMs can totally avoid the collisions from hidden nodes even in the worst case. However, in practice, interference from hidden nodes is not always that serious. This suggests that the estimated CSRs in PhIMs sometimes become larger than the correct value of CSR. An over-estimated CSR will cause the explosion problem and some contending nodes will miss the transmission opportunities. As a result, the effective channel utilization rate, which has a positive correlation with the system throughput, will be decreased.

In this paper, we propose an effective method to improve the network throughput performance by controlling the interference from neighbor nodes including hidden nodes. Our main contributions in this paper are summarized as bellow:

Firstly, we analyze the characteristics of the pre-configuring operations in WSNs. Based on such operations, we propose a data collection scheme with distinct directivity. We validate of this directional transmission scheme on the premise of complete data collection.

Secondly, we analyze collisions in WSNs and propose a space-time based medium access mechanism, which explores user diversity both in space and time domains. By fully taking advantage of the directional transmission strategy, we propose a new method of calculating CSRs for the proposed channel access mechanism to significantly reduce or even totally eliminate in some cases collisions from both neighbor nodes and hidden nodes.

Thirdly, we conduct simulations to evaluate the performance of our proposed solution, which was compared to the classic IEEE 802.11 series protocols and ones with enhanced CSRs. We analyze the throughput and the packet loss rate, the key parameters that determine the data collection efficiency. In order to cover scenarios with different settings, we evaluate the performance of our proposed solution in networks with different topologies, including the regular latticed case and random deployed ones.

This paper is organized as follows. In Section 2 we analyze the channel access characteristics in WSNs and propose a new directional data transmission strategy. A space-time based random access method is proposed in Section 3. In Section 4, we evaluate the performance of the proposed method through simulations. In Section 5 we conclude this paper.

## 2. Data Collection Strategy with Directional Transmission

Communications in WSNs are quite different from general wireless networks in that the data flow is quite directional, i.e., either from the sink node to end nodes or vice versa. In this section, we analyze these properties ofa WSNs and propose our new data collection strategy with directional transmission characteristics.

### 2.1. Pre-Configuring Operation in WSNs

In WSNs, a sink node is the controller of the whole network by sending commands to other sensors, including monitoring the sensors’ status, broadcasting commands to sensors, etc. This task is called the run-up phase. The second task for WSNs is to collect data from all the sensors where data will be sent to the sink node using intermediate relay sensor nodes. This task is called the data-collection phase. Controlled by the sink node, the WSN can either be in the run-up phase or data-collection phase.

The run-up phase of the WSN happens during the network deployment process when some simple interactions happen between the sensors and the sink node. For example, the sink node will unicast, multicast or broadcast commands to sensors and then the sensors will report their state information back to the sink node, i.e., their position, battery, routing information, etc. to the sink node. By checking the information above, the sink node can ensure the network connectivity while sensors can update their information to the sink node.

The data-collection phase happens when an event is detected by sensors so that the sensors need to send data back to the sink node. During this period of time, a large number of nearby sensors will simultaneously attempt to get access to the channel in order to transmit the data to the sink node. Since CSMA/CA is not good at supporting large users contending for channel access, the above case will result in serious collisions among the sensors which significantly decreases network throughput and introduce more delay.

Considering those characteristics of communications in WSNs, it is necessary to introduce centralized control, i.e., making some pre-configurations to sensors and introducing some rules to define specific behaviours of sensors especially during the run-up phase. With the pre-configuration in the run-up phase, data transmissions in the data-collection phase can be well controlled by avoiding extreme congestions, so that collisions are able to be suppressed.

### 2.2. Directional Transmission Model

Directional data forwarding is a typical feature of communications in WSNs because the data flow in WSNs is always directional, i.e., it is either from sensors to the sink node or vice versa. Considering the pre-configuring operations during the run-up phase, the property of directional data transmissions in WSNs can be further utilized and the interference among nodes can be better mitigated. Next, we focus on the analysis of the directional transmission model.

**Figure 1:**
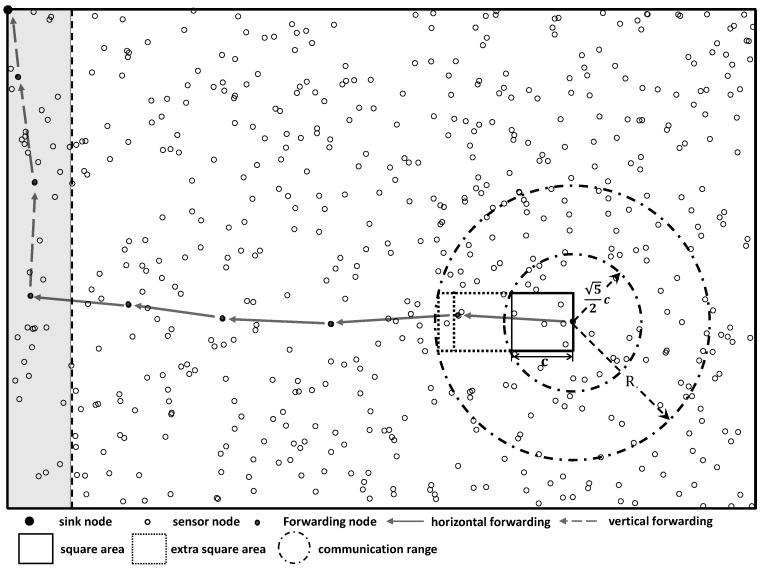
Directional data forwarding topology for a high density distributed wireless sensor network.

We start with the physical model of a sensor network. As shown in Figure 1, we consider a sensor network which includes *n* wireless sensor node $V=\{v_{1},v_{2},\cdot \cdot \cdot ,v_{n}\}$ and a sink node *s*. We use a random extended network model [13] by assuming that sensor nodes are uniformly deployed in a rectangular region with the size of a×b.

We denote the location of the sensor node $v_{t}$ in the network by

(1) $$v_{t}\frac{def}{\;}\left(x_{t},y_{t}\right)$$

where $x_{t}$ and $y_{t}$ represent the location coordinates. We denote the distance between $v_{t}$ and $v_{r}$ as the euclidean space $\left|\right|v_{t}-v_{r}\left|\right|$ and the communication range as *R*, which is associated with the Signal to Interference plus Noise Ratio (SINR). If $\left|\right|v_{t}-v_{r}\left|\right|\leq R$, data transmission from $v_{t}$ to $v_{r}$ will be successful. We denote the link as $l(v_{t},v_{r})$ and express the links in the network as Equation (2).

(2) $$L=\{l\left(v_{t},v_{r}\right)\;|\;||v_{t}-v_{r}||\leq R,v_{t},v_{r}\in V\}$$

The sink node is placed at the upper-left corner with the coordinate (0,0). Thus, the data collection process is divided into two parts: horizontal forwarding and vertical forwarding. During the horizontal forwarding process, data goes from the right to the left which is shown as the data forwarding in the white area of the Figure 1, and for the vertical forwarding, data goes from bottom to top which is shown as the data forwarding in the gray area of the Figure 1. The horizontal forwarding and vertical forwarding processes are separated by different methods and resources including channels and time slots, etc. Next we construct the directional data forwarding in the horizontal forwarding process as an example since the vertical forwarding process can be done in the same way.

The property of directional data transmission in the horizontal forwarding process is analyzed by introducing the link constraint between sensor nodes. The link constraint is that a link from $v_{t}$ to $v_{r}$ is available, if and only if the projected length of $l(v_{t},v_{r})$ in the vertical axis is smaller than $\sqrt{\frac{a\cdot b\log n}{2\cdot n}}$. Denoting $\left|\right|v_{t}-v_{r}{\left|\right|}_{y}$ as the projected length of $l(v_{t},v_{r})$ in the vertical axis, the links which satisfy the above link constraint can be expressed as Equation (3).

(3) $$L^{*}=\{l\left(v_{t},v_{r}\right)\;|\;v_{t},v_{r}\in V,||v_{t}-v_{r}{\left|\right|}_{y}\leq \sqrt{\frac{a\cdot b\cdot \log n}{2\cdot n}}\}$$

### 2.3. Model Validity Analysis

$L^{*}$ in our directional transmission strategy is a subset of *L*. In order to make sure all the data from the sensor nodes can be sent to the gateway nodes by $L^{*}$ out of *L*, we need to ensure the network is completely connected based on the above model.

As shown in Figure 1, by setting the coordinate of a sender $v_{t}$ as the midpoint of the right side, a square area is created with the side length of *c*. In a uniformly distributed network with the size of a×b, the probability of a random deployed sensor node within the above square area is equal to $\frac{c^{2}}{a\cdot b}$. Thus, in a *n*-node system, the probability of having an empty square area can be calculated by

(4) $$p_{n}={\left(1-\frac{c^{2}}{a\cdot b}\right)}^{n}$$

where

(5) $$c=\sqrt{\frac{2\cdot a\cdot b\cdot \ln n}{n}}$$

Because 1-x≤exp(-x), we have

(6) $$p_{n}\leq \exp\left(\frac{-n\cdot c^{2}}{a\cdot b}\right)=\frac{1}{n^{2}}$$

and

(7) $$\sum_{n=1}^{\infty }p_{n}<\infty$$

According to the Borel-Cantelli lemma, there is at least one sensor node located in the square area for sufficiently large *n*.

As shown in Figure 1, if the communication range *R* is larger than $\frac{\sqrt{5}}{2}\cdot c$, at least one sensor node can be selected as a receiver by the sender $v_{t}$ to establish a link. Because $v_{t}$ is in the middle of the right side of the square, the projected length of the link in the vertical axis is smaller than $\frac{c}{2}$. Forwarding data from all the sensor nodes to the gateway nodes through the links in set $L^{*}$ is feasible. As the number of sensor nodes located in the square area is a binomial random variable with parameters $\left(\frac{c^{2}}{a\cdot b},n\right)$, the availability of links in the square area is guaranteed. As shown in Figure 1, if the communication range *R* is large enough, which is very common in practice, the sender $v_{t}$ can select receivers from other nearby square areas. In this situation, more links to do the data forwarding are available.

Now, we define $\rho =\frac{n}{a\times b}$, where *ρ* represents the sensor density of a WSN. According to Equation (5), a smaller square area can be constructed by larger *ρ*. In a dense WSN, our proposed directional data forwarding scheme for both the horizontal and vertical cases can be modelled as a problem of bunch of parallel-arranged lines with certain offsets which decrease significantly as a function of the sensor nodes density. For the scenarios that a WSN has a regular latticed topology, the data forwarding links are in parallel to each other with no offsets and can be expressed as c=0.

## 3. A Space-Time Based Random Access Mechanism

### 3.1. Collisions Analysis

The set of nodes that have collisions with the data transmission from $v_{t}$ to $v_{r}$ can be defined as concurrent nodes. Because there are multiple concurrent nodes, the concurrent nodes can be further grouped as concurrent senders, which are the nodes that are sending data to their corresponding receivers and concurrent receivers that are passively responding to the corresponding senders for handshaking purpose.

Base on the literature review on the Carrier Sense Range (CSR), the proposed CSR based schemes to protect the on-going transmission from being collided by concurrent nodes, can be expressed as follows:

(8) $$CSR=\left(K+\beta \right)\cdot d_{max}$$

where $d_{max}$ is the maximum link length in the network. *K* is a coefficient calculated from the signal propagation loss model [4,11]. All the concurrent nodes including both concurrent senders and receivers are considered in the model above. The power levels of those concurrent nodes are accumulated at the receiver as interference and *K* is determined to make sure that the lower bound of the signal to interference plus noise ratio (SINR) at the receiver is larger than a given threshold.

**Figure 2:**
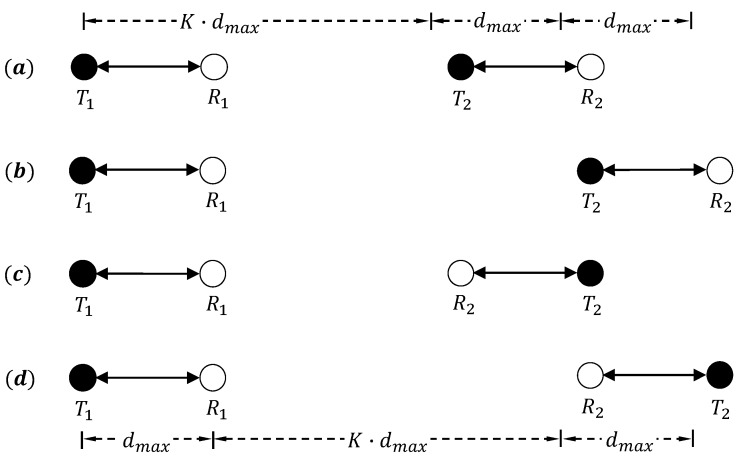
Diagram of interference from concurrent senders on the basis of coefficient *K*.

$K\cdot d_{max}$ defines the range which eliminates collisions from concurrent nodes. However, it does not differentiate the roles of the concurrent nodes involved, if they are the concurrent senders or receivers, or their position information. In order to make a receiving node maintain an ideal SINR according to $K\cdot d_{max}$, an extra range is needed to better separate the transmissions between concurrent nodes as sources of interference and the receiver of interest. The above problem through the two pairs of communications $\left(T_{1},R_{2}\right)$ and $\left(T_{2},R_{2}\right)$ is illustrated in Figure 2. The carrier sense range is initially defined by $K\cdot d_{max}$. As shown in Figure 2a, $T_{1}$ and $T_{2}$ are out of each other’s carrier sensing range. As a result, the sending activity of $T_{2}$ can not be stopped by the sending node $T_{1}$. However, the noise from $T_{2}$ may collide with $R_{1}$’s received signal. This problem can be solved through extending CSR by $d_{max}$ as shown in Figure 2b. $R_{1}$’s signal may also collide with the signal between $T_{2}$ and $R_{2}$ in Figure 2c because of the directional communication introduced. In this case, CSR needs to be extended by another $d_{max}$ to eliminate the collisions as shown in Figure 2d. An extra extension of CSR is defined as *β*. According to the existing literature, the value of *β* is often larger than 2.

According to the above analysis, the network topology, the positions of the concurrent senders and receivers, and the data transmission direction can jointly determine the value of the carrier sense range. With the directional transmission strategy, the positions of the concurrent nodes and the data transmission directions can be well controlled to minimize collisions. A new random medium access scheme is further proposed to allow multiple users to access the channel from both space and time domains.

### 3.2. Space-Time Based Random Access Mechanism

We define the coordinate system as follows. The data forwarding direction is denoted as the *x*-axis, and then the sensor nodes are grouped as *B*-group along the *x*-axis according to Equation (9).

(9) $$B_{i}=\left\{v_{t}\;|\;x_{t}\in \left[i\cdot D,i\cdot D+D\right)\right\},i=0,1,2,\cdot \cdot \cdot$$

where *D* is the width of *B*-group. Based on the grouping rules in Equation (9), the sensor nodes are divided into Δ groups, which are named as the *G*-group according to Equation (10):

(10) $$G_{k}=\underset{i=0}{⋃}B_{i\cdot \Delta +k},k=0,1,\cdot \cdot \cdot ,\Delta -1$$

The data collection time is divided into Ts and sensor nodes in $G_{k}$ can only access the channel during the time interval of $T_{k}$.

(11) $$T_{k}=\underset{i=1}{⋃}\left[i\cdot \Delta -k-1,i\cdot \Delta -k\right)\cdot T$$

### 3.3. CSR for Space-Time Based Random Access Mechanism

According to the system model above, concurrent senders can only exist in the same *G*-group based on the fact that only the nodes belonging to the same *G*-group can access the channel at the same time interval of *T*. The concurrent senders are divided into two categories based on whether they belong to the same *B*-group or not. Next, the CSRs are designed for our proposed random access mechanism to eliminate collisions and its performance is evaluated.

#### 3.3.1. Eliminating Interference from the Same B-Group

**Figure 3:**
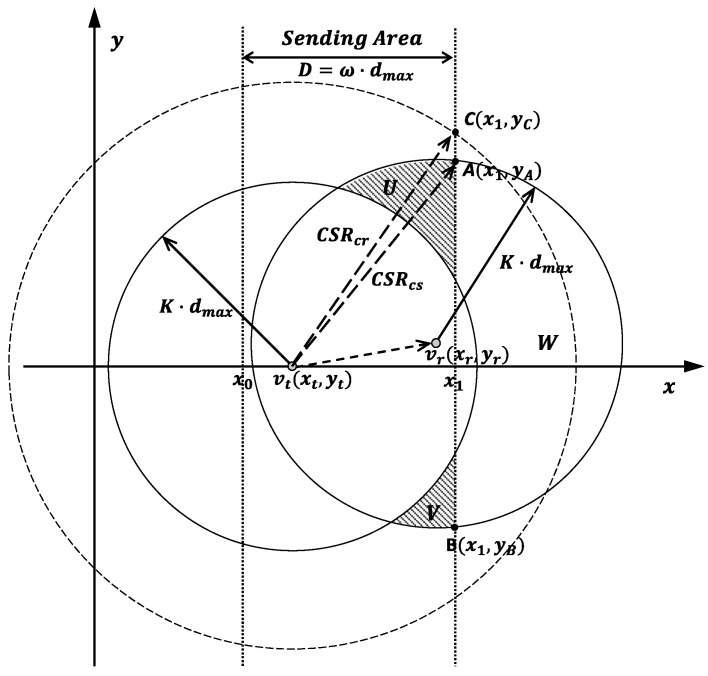
Diagram of interference from concurrent senders in the same B-group.

The interference from concurrent senders in the same B-group is analyzed through Figure 3. In the figure, node $v_{t}$ with coordinate $\left(x_{t},y_{t}\right)$ is sending data to node $v_{r}$ with coordinate $\left(x_{r},y_{r}\right)$. The carrier sense range is firstly initialized into $K\cdot d_{max}$, and the carrier sense areas of $v_{t}$ and $v_{r}$ are expressed as two circles shaped by Equations (12) and (13).

(12) $${\left(x-x_{t}\right)}^{2}+{\left(y-y_{t}\right)}^{2}=K^{2}\cdot d_{max}^{2}$$

(13) $${\left(x-x_{r}\right)}^{2}+{\left(y-y_{r}\right)}^{2}=K^{2}\cdot d_{max}^{2}$$

The concurrent nodes are in a susceptible area, which contains the carrier sense area of $v_{r}$ expected for the carrier sense area of $v_{t}$. As shown in Figure 3, such a susceptible area is divided into three parts labeled as *U*, *V* and *W* by the sending area with the width of *D*. We denote the left and right boundaries of the sending area as $L_{0}:x=x_{0}$ and $L_{1}:x=x_{1}$. According to the definition of B-group in Equation (9) and the time assignment in Equation (11), if nodes are located in a band region with the width of *D* ($D=x_{1}-x_{0}$) access channel, the nodes in the adjacent regions are not allowed to access the channel. Thus, the distribution of the concurrent nodes is significantly affected by the configuration of the sending area.

##### Concurrent Senders Elimination

According to Figure 3, concurrent senders will only stay in the area of U⋃V. In order to eliminate the interference from these concurrent senders, the carrier sense area is extended to cover the area of U⋃V. By substituting $x=x_{1}$ to Equation (13) and then working on the equation, we get the coordinates of the two intersections $A(x_{1},y_{A})$ and $B(x_{1},y_{B})$ as shown in Figure 3. From the above figure, we can get $y_{A}=y_{B}$ when we have $y_{t}=y_{r}$. It can be easily proved that by setting:

(14) $$CSR_{cs}\geq \sqrt{{\left(x_{1}-x_{t}\right)}^{2}+\max\left\{y_{A}^{2},y_{B}^{2}\right\}}$$

the concurrent senders of the transmission from $v_{t}$ to $v_{r}$ can be totally eliminated. Because $v_{t}\in sending\;area$, it has $x_{t}\in \left[x_{0},x_{1}\right]$. Thus, we get $x_{1}-x_{t}\leq D$. According to the equation of $|y_{t}-y_{r}|\leq \frac{c}{2}$ from the previous section, we have $|y_{A}\left|,\right|y_{B}|\leq K\cdot d_{max}+\frac{c}{2}$. Based on the above, by setting:

(15) $$CSR_{cs}\geq \sqrt{D^{2}+{\left(K\cdot d_{max}+\frac{c}{2}\right)}^{2}}$$

the collisions to an on-going transmission generated by any sender in the sending area can be eliminated.

Here, $d_{max}$ is used to evaluate *D* and *c* by defining $\omega =\frac{D}{d_{max}}$ and $\theta =\frac{c}{d_{max}}$. Thus, Equation (15) can be rewritten as

(16) $$CSR_{cs}\geq \sqrt{\omega^{2}+{\left(K+\frac{\theta }{2}\right)}^{2}}\cdot d_{max}$$

##### Concurrent Receivers Elimination

A sender which is out of the carrier sense range of $v_{t}$ is denoted as $v_{tt}\left(x_{tt},y_{tt}\right)$. By defining CSR according to Equation (16), it can have

(17) $$y_{tt}\geq \left(K+\frac{\theta }{2}\right)\cdot d_{max}$$

Then, $v_{tt}$’s receiver is denoted as $v_{rr}\left(x_{rr},y_{rr}\right)$. According to the directional data forwarding property, $y_{rr}$ has

(18) $$y_{rr}\in \left[x_{rr}-\frac{\theta }{2}\cdot d_{max},x_{rr}+\frac{\theta }{2}\cdot d_{max}\right]$$

By substituting Equation (17) to Equation (18), we get

(19) $$y_{rr}\geq K\cdot d_{max}$$

Because $y_{rr}$ is smaller than the upper bound of the carrier sense range of $v_{r}$, $v_{rr}$ may become the concurrent receiver of the transmission from $v_{t}$ to $v_{r}$. Thus as shown in Figure 3, we extend the CSR to get rid of these potential concurrent receivers according to

(20) $$CSR_{cr}\geq \sqrt{\omega^{2}+{\left(K+\theta \right)}^{2}}\cdot d_{max}$$

$y_{tt}$ can be controlled as Equation (21) below

(21) $$y_{tt}\geq \left(K+\theta \right)\cdot d_{max}$$

Then it has

(22) $$y_{rr}\geq \left(K+\frac{\theta }{2}\right)\cdot d_{max}$$

which is out of the carrier sense range of $v_{r}$.

##### CSR Configuration

By comparing Equations (16) and (20) to the basic carrier sense range $K\cdot d_{max}$, concurrent nodes can be totally eliminated if CSR is set up according to

(23) $$CSR=\sqrt{\omega^{2}+{\left(K+\theta \right)}^{2}}\cdot d_{max}$$

As shown in Equation (23), the safe carrier sense range is determined by three parameters *K*, *θ* and *ω*. According to our analysis of the above, *K* is the lower bound of CSR while *θ* indicates the maximum data forwarding offset which is affected by the network density according to our analysis in Section 2. Thus, the only variable is *ω* which indicates the width of *B*-group *D*. Next, we focus on the configuration of *D*.

According to Equation (23), CSR increases as a function of *D*, suggesting that a larger *B*-group leads to a lower channel utilization rate, which corresponds to a lower network throughput. From this point of view, a smaller *B*-group is preferred. However, the smaller *B*-group means that fewer nodes can be included in a single group. If *B*-group becomes small, some areas may become empty with no sensors, indicating that the channel utilization rate will also be decreased. This becomes worse if only a few nodes need to transmit data and the effective channel utilization rate will decreased significantly.

In order to get a balanced size of *B*-group, *D* is set as the minimum link length in the network $d_{min}$ with the following benefits.

- Firstly, $d_{min}$ is a crucial factor constrained by the network layer configuration in multi-hop data collection networks and it can be calculated during the run-up phase.
- Secondly, a large number of short links on the data transmission path leads to a longer time of data forwarding. It will not only lead to channel contention but also increase the data forwarding delay, thus $d_{min}$ is always set as large as possible while considering the reliability of data reception. Thus, by setting $D=d_{min}$, *B*-group can contain a sufficient number of nodes sharing the channel.
- Thirdly, we set $D=d_{min}$ to ensure that data will be forwarded to other *B*-groups during the following *T* time slots. This structure can effectively increase the channel utility rate during a low traffic period.

#### 3.3.2. Eliminating Interferences Between B-Groups

The distance between two concurrent senders belonging two different *B*-groups is larger than (Δ-1)·D. According to the analysis above, there is one and only one receiver associated with the two senders. By denoting the maximum communication range in *x*-axis as $d_{x\_max}$, the minimum distance between the receiver and the interfering sender is larger than $\left(\Delta -1\right)\cdot D-d_{x\_max}$. According to the conclusion in Equation (8), the safe range between a receiver and a interfering sender is larger than $K\cdot d_{max}$. We get

(24) $$\left(\Delta -1\right)\cdot D-d_{x\_max}\geq K\cdot d_{max}$$

We have $d_{max}\geq d_{x\_max}$. By substituting it to Equation (24) and then doing some transpositions, we can get

(25) $$\Delta \geq \frac{\left(K+1\right)\cdot d_{max}+D}{D}$$

During the groups of sensor nodes taking turns accessing the wireless channel as Equations (10) and (11) indicated, the channel utility rate will be decreased by the oversized Δ. Thus, in our proposed method, we can set it according to the following for any determined *B*-group width *D*.

(26) $$\Delta =\lceil \frac{\left(K+1\right)\cdot d_{max}+D}{D}\rceil$$

The idea of the above is to totally eliminate collisions from concurrent senders belonging to different *B*-groups.

## 4. Performance Evaluation

The performance of the proposed schemes is evaluated through simulations using OMNET++ [14]. The classic protocols (IEEE 802.11x [15], IEEE 802.15.4 [16]), and other channel access mechanisms with enhanced CSRs [4] are compared in this section. Simulations are conducted with different network scales. Along with the increasing of the network scales, the number of neighbor nodes and hidden nodes increase. In our simulations, the performance of different data collection strategies is evaluated by analyzing the effects of interference from neighbor nodes and hidden nodes on the network performance of throughput, collision rate and frame loss rate, which are all key factors determining the data collection efficiency in WSNs. In addition, in order to cover the diversity of WSN applications, we consider two typical topologies, i.e., the random topology and the latticed topology.

### 4.1. Simulation Configurations

#### 4.1.1. PHY and MAC Layer Configurations

The related PHY and MAC layer parameters are listed in Table 1 based on the IEEE 802.11b standard [15] which supports a longer distance than that of IEEE 802.15.4. Since the backoff process is universal in all the IEEE 802.11x and IEEE 802.15.4 standards, the proposed algorithm and its performance apply to all the backoff based medium access schemes in IEEE 802.11x and 802.15.4 networks.

#### 4.1.2. Path Loss Model Configuration

In the simulations, the signal attenuates during the propagation in the form of the well known simple path loss model [17] which is expressed in Equation (27).

(27) $$PL={\left(\frac{\lambda }{4\pi }\right)}^{2}d^{-\alpha }$$

In Equation (27), PL is the pass loss, and *λ* is the wavelength, *d* is the transmitter-receiver distance and *α* is the path loss exponent. For WiFi applications, *α* is often set from 2 to 4. Because the value of *α* does not affect the final simulation results, we set α=4 in our simulations.

#### 4.1.3. Topology Configuration

The WSN topology is an important factor in the simulations to make sure it comprehensively represents the real settings in WSNs. Since the corresponding communication range is around 80 m for α=4, we set up an area of 400 m × 400 m with 100 sensors using different deployment schemes. In our simulations, we evaluate the proposed algorithms in networks with different settings.

#### 4.1.4. Contrast Simulations

In order to comprehensively evaluate our proposed data collection method, we make five types of simulations with different kinds of data collection methods listed as below.

- IEEE802.11—This is the traditional data collection method in WSNs, where all sensors are implemented with the IEEE 802.11 protocol.
- PhIM—This is a data collection method proposed in [4] based on IEEE 802.11 with a larger CSR to totally eliminate collisions from hidden nodes and with a low signal receiving sensitivity.
- PhIM_Est—Because collisions from hidden nodes are totally eliminated in “PhIM”, the estimation-based backoff algorithm is used in this algorithm to further decrease collisions from neighbor nodes. Thus, this is a data collection method with the classical estimation-based backoff and idle sense algorithm [2].
- ST-RAM—This is our proposed data collection method with updated CSRs by utilizing the directional data forwarding characteristics in WSNs.
- ST-RAM_Est—This is our proposed data collection method working together with the estimation-based backoff algorithm.

Next, the performance of the above five data collection methods is compared in terms of throughput and frame loss rate with the above two kinds of topologies.

### 4.2. Throughput Performance

**Figure 4:**
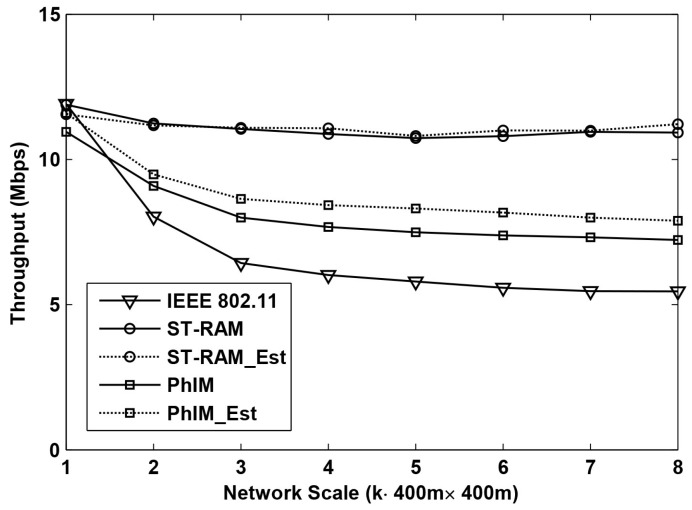
Throughput performance of different data collection methods for random deployed networks with different scales.

**Figure 5:**
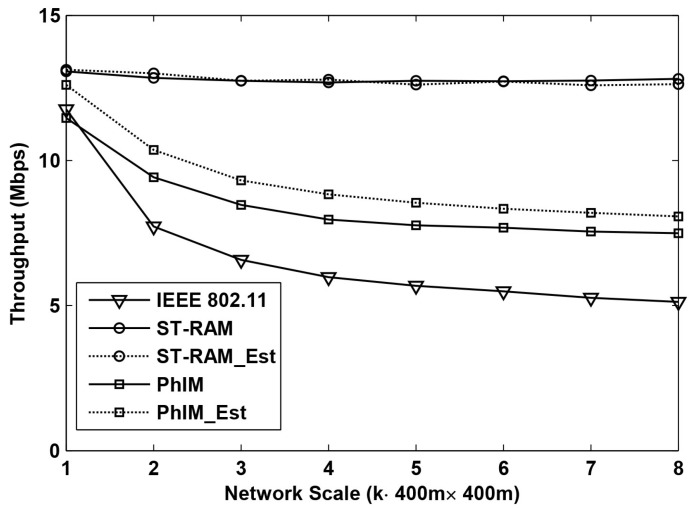
Throughput performance of different data collection methods for lattice deployed networks with different scales.

Throughput is one of the most important performance indexes in WSNs. Figure 4 and Figure 5 show the results of the throughput when using different data collection methods in the networks with different scales. The network scales ranging from 400 m × 400 m to 400 m × 3200 m are presented in the figure which is indicated by the x-axis. The y-axis is the average throughput of 400 m × 400 m area on average.

Figure 4 shows the throughput as a function of the network scale with random topologies. From the figure we can see that when the network scale is small, the throughput of different data collection methods is similar but when the network scale is increased, the throughput become very different.

Compared to the five different data collection methods, the throughput of “IEEE802.11” decreases much faster than of the other schemes. This is because the number of hidden nodes of an on-going communication increases exponentially along with the increase of the network scale. As a result, interference from hidden nodes severely decreases the throughput of “IEEE802.11”. The farther a hidden node is, the weaker interference it can cause. Thus, with the increase of the network scale, the accumulated interference from hidden nodes tends to be stable which makes the throughput of “IEEE802.11” stable at around 5 Mbps when *k* is larger than 4.

The throughput of “PhIM” is much higher than that of “IEEE802.11”, because the interference from hidden nodes is significantly suppressed by setting large CSR. However, with the increase of the network scale, the throughput of “PhIM” can only reach a certain level. It is because a larger CSR not only suppresses the number of the hidden nodes but it also increases the number of the neighbor nodes. The number of the neighbors will increase as a function of the extension of the network coverage. A larger number of neighbor nodes with more serious channel contention will also decrease the throughput.

The throughput decrease resulting from the neighbor nodes can be relieved by “PhIM_Est” which can be easily verified by the two curves labeled with “PhIM” and “PhIM_Est” in the figure. It is because the estimation-based backoff algorithm which is applied in “PhIM_Est” can effectively reduce collisions from the extremely large number of neighbor nodes.

In all the simulations, our proposed data collection method “ST-RAM” performs the best with the most stable throughput even for the cases of increasing the network scale. This is because our proposed solution has the following three advantages: Firstly, collisions from the hidden nodes are totally eliminated by our new proposed CSR. Secondly, the value of CSR is cut down by sufficiently utilizing the communication rules in WSNs which significantly improves the effective channel utilization. Thirdly, sensors are allowed to access the channel in different time intervals in our proposed method which further decreases collisions from neighbor nodes.

Figure 4 shows that the throughput of “ST-RAM” and that of “ST-RAM_Est” is about the same. This is reasonable because the number of concurrent neighbor nodes in our proposed method is significantly decreased by the time division multiple access mechanism so that the number of contending neighbors is only around a dozen for our settings. In this case, collisions from neighbor nodes are so low that the possibility of collisions that can be avoided by the estimation-based backoff algorithms is very small. This conclusion can be verified according to the results in [3].

The throughput of different data collection methods in latticed topology is shown in Figure 5. By comparing the results in Figure 4 and Figure 5 we can find that the throughputs of “IEEE802.11”, “PhIM” and “PhIM_Est” in the random topology are almost the same as that of the latticed topology case. This is because the topological structure is not taken into consideration in designing the value of CSR by the above methods. However, in our proposed method, the position relationship among the sensors plays an important role in obtaining better CSRs. According to Equation (23), we can find that the CSR in the latticed topology can be further optimized by substituting θ=0 which is proved in Section 2. This conclusion is verified in Figure 5 where the throughput of “ST-RAM” and “ST-RAM_Est” in the latticed topology are obviously higher than that in the random topology.

### 4.3. Collision Rate and Frame Loss Rate

Collision rate is another important performance parameter in WSNs. The MAC layer protocol of WSNs requires that if collision happens the frame will be retransmitted until it is successfully received or reaches the maximum number of retransmissions tries. This retransmission mechanism is applied to avoid endless retransmissions over very bad quality links. Once the maximum number of retransmissions is reached, the node will drop the frame. This may trigger an upper layer retransmission during which a new transmission route will be generated. However, the frame loss may be because of the interference from other sensors. In this case, the upper layer retransmission will be also triggered; however, this does not make any sense, because congestions in WSNs are always within a region. However, during this process, the new transmission route establishment process will cost a lot of time that is tens or hundred times of an ordinary frame transmission in the MAC layer. This will cause a severe throughput decrease on the basis of the results we presented in the previous section.

**Figure 6:**
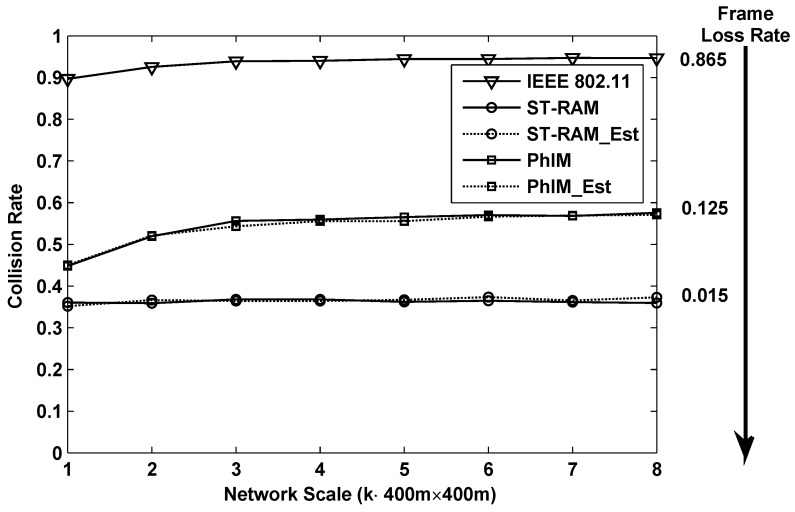
The collision rate and frame loss rate of different data collection methods for random deployed networks with different scales.

**Figure 7:**
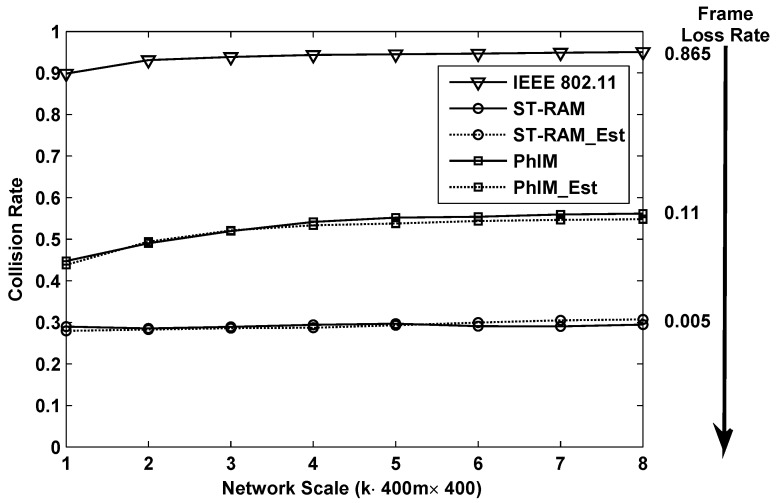
The collision rate and frame loss rate of different data collection methods for lattice deployed networks with different scales.

Figure 6 and Figure 7 show the results of the collision rate in using of different data collection methods in the networks with different scales. Results shown in Figure 6 are for the case in the random topology. It can be seen that, being affected by the serious collisions from hidden nodes, “IEEE802.11” has the highest collision rate which is larger than 0.95 in large-scale networks. While eliminating the collisions from hidden nodes and bringing a larger number of neighbor nodes, the collision rates of “PhIM” and “PhIM_Est” are close and both approach 0.6 in large-scale networks. Our proposed methods “ST-RAM” and “ST-RAM_Est” have the lowest collision rate by simultaneously decreasing the collisions from hidden nodes and contending neighbor nodes.

According to the IEEE 802.11 protocol, we set the maximum number of retransmissions as 4 and simulate the corresponding frame loss rate in networks with different scales. The approximate frame loss rates in large scale networks, where the frame loss rate becomes stable, are labeled on the right side of Figure 6. An extremely large amount of time will be wasted on establishing a new route in “IEEE802.11” because of its 86.5% frame loss rate. The frame loss rate in “PhIM” is acceptable which is around 12.5% and our proposed “ST-RAM” can achieve extremely low packet loss rate which is less than 2%.

The collision rate and frame loss rate of different data collection methods in the latticed topology is shown in Figure 7. Our proposed method “ST-RAM”, the frame loss rate can even be less than 1%.

### 4.4. Performance in Networks with Different Densities

**Figure 8:**
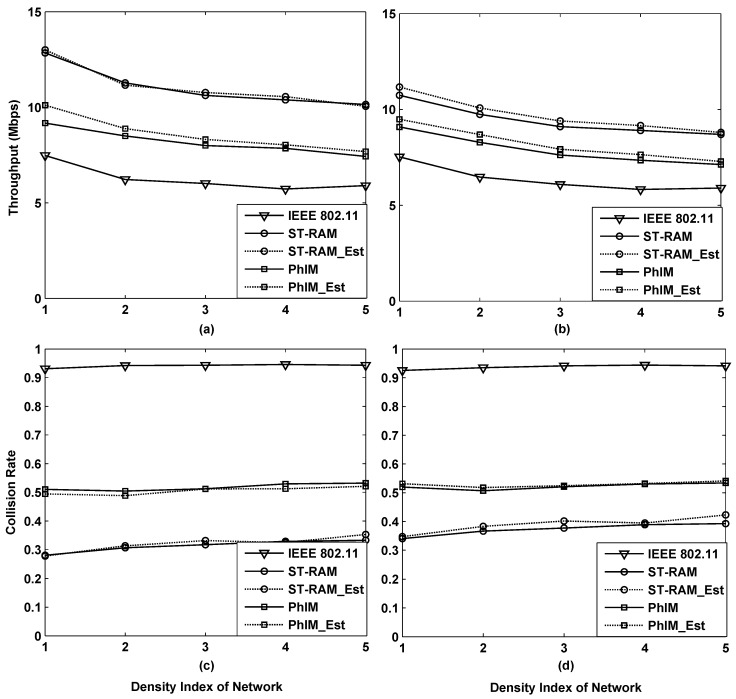
Throughput performance of different data collection methods for lattice deployed networks (**a**) and the random deployed networks (**b**); Collision rate of different data collection methods for lattice deployed networks (**c**) and random deployed networks (**d**).

In above simulations, the network topologies are all constructed in the unit area where 100 sensors locate in 400 m × 400 m. In order to study the performance of different data collection methods in networks with other densities, the network with the scale of 400 m × 800 m are constructed and new simulations where the network densities are 1 to 5 times as much as 100/(400 m × 400 m) are configured. Figure 8 shows the simulation results where four subgraph respectively represents the throughput and collision rate of different data collection methods for lattice deployed networks and random deployed networks.

In the figure, the density index equals the simulated network density divided by 100/(400 m × 400 m). Figure 8a,b show that the throughput performances of different data collection methods all decrease along with the increase of the network density, and tend to be stable when the network densities are extremely large. It works both in lattice deployed networks and the random deployed networks. This is mainly because the interferences from hidden nodes in IEEE 802.11 become more and more serious, until the energies from additional hidden nodes are negligible compared to the accumulated interferences. Besides, the significant increasing number of neighbor nodes is another main cause of the throughput decrease. Similar results can be obtained from the collision rate performance in Figure 8c,d. By comparing the performance of different data collection methods in Figure 8, it can be found that our proposed method “ST-RAM” achieves the best and the most stable throughput and collision rate.

## 5. Conclusions

In IEEE 802.11 based wireless networks, throughput is significantly affected by collisions and interference particularly in widely spread and densely deployed WSNs. Mitigating interference and reducing these collisions is recognised as a research challenge. In this paper, the characteristics of communications in WSNs were analyzed and a directional data transmission strategy by sufficiently utilizing these communication features was proposed. Based on this directional strategy, a space-time based random access method for WSNs was further provided.

To evaluate the performance of our proposed method, comprehensive simulations were performed to compare with other methods for WSNs reported in the literature. Simulation results show that our proposed method can achieve the frame loss rate of less than 2% in large scale WSNs and the average network throughput can be effectively improved to 2 times that of the other channel access schemes in WSNs.

## Figures and Tables

**Table 1:** PHY layer and MAC layer parameters used in simulations.

Parameters	Value	Parameters	Value
Channel Bit Rate	11 Mbps	Payload Length	2304 Byte
Slot Time (ST)	20 μs	MAC Header	224 bit
SIFS	10 μs	RTS	160 bit
DIFS	50 μs	CTS	112 bit
PHY Header	192 bit	ACK	112 bit
